# High serum resistin associates with intrahepatic inflammation and necrosis: an index of disease severity for patients with chronic HBV infection

**DOI:** 10.1186/s12876-016-0558-5

**Published:** 2017-01-07

**Authors:** Zhongji Meng, Yonghong Zhang, Zhiqiang Wei, Ping Liu, Jian Kang, Yinhua Zhang, Deqiang Ma, Changzheng Ke, Yue Chen, Jie Luo, Zuojiong Gong

**Affiliations:** 1 Department of Infectious Diseases, Hubei University of Medicine, Shiyan, China; 2 Institute of Biomedicine, Hubei University of Medicine, Shiyan, China; 3 Institute of Wudang Chinese Medicine, Hubei University of Medicine, Shiyan, China; 4 Department of Neurology, Taihe Hospital, Hubei University of Medicine, South Renmin Road. 32, 442000 Shiyan, Hubei China; 5 Department of Infectious Diseases, Renmin Hospital of Wuhan University, Zhangzhidong Road. 99, 430060 Wuhan, China

**Keywords:** Resistin, Proinflammatory cytokines, Hepatitis B, Liver cirrhosis, Liver failure

## Abstract

**Background:**

Studies have revealed that resistin plays a role as an intrahepatic cytokine with proinflammatory activities. This study investigated the association between serum resistin and fibrosis severity and the possible marker role of resistin in the inflammatory process of chronic hepatitis B.

**Methods:**

In this study, 234 subjects with HBV infection were retrospectively selected, including 85 patients with chronic hepatitis B (CHB), 70 patients with HBV-related liver cirrhosis (LC-B), and 79 patients with HBV-related liver failure (LF-B). Serum levels of resistin, IL-1, IL-6, IL-17, IL-23, TNF-α, and TGF-β1 were assayed by ELISA. Demographic and clinical characteristics of patients were extracted from clinical databases of Taihe Hospital, Hubei University of Medicine, including serum levels of alanine aminotransferase (ALT), aspartate aminotransferase (AST), total bilirubin (TBil), and liver stiffness (LS).

**Results:**

All the selected patients with HBV infection showed significantly increased levels of serum resistin, which was rarely detectable in the healthy controls. Serum resistin levels in patients with CHB, LC-B, and LF-B were 4.119 ± 5.848 ng/mL, 6.370 ± 6.834 ng/mL, and 6.512 ± 6.076 ng/mL, respectively. Compared with the CHB group, patients with LC-B or LF-B presented with significantly higher serum levels of resistin (*p* < 0.01). On the other hand, all of the enrolled patients had high serum levels of IL-1, IL-6, IL-17, TNF-α, and TGF-β1, but not IL-23. Interestingly, serum levels of resistin was significantly positively correlated with serum levels of TGF-β1 in LC-B patients (*R* = 0.3090, *p* = 0.0290), with IL-17 in LC-B (*R* = 0.4022, *p* = 0.0038) and LF-B patients (*R* = 0.5466, *p* < 0.0001), and with AST (*R* = 0.4501, *p* = 0.0036) and LS (*R* = 0.3415, *p* = 0.0310) in CHB patients.

**Conclusions:**

High serum resistin associates with intrahepatic inflammation and necrosis and may be used as an index of disease severity for patients with chronic HBV infection.

**Electronic supplementary material:**

The online version of this article (doi:10.1186/s12876-016-0558-5) contains supplementary material, which is available to authorized users.

## Background

HBV infection is the most common cause of chronic liver injury and a major cause of liver cirrhosis, hepatocellular carcinoma, and liver failure [1]. Liver failure is a severe clinical syndrome characterized by jaundice, ascites, hepatic encephalopathy, and a bleeding tendency due to impairment of liver function, leading to poor prognosis [2]. According to World Health Organization estimates, approximately 240 million people around the world suffer from chronic HBV infection (http://www.who.int/hiv/pub/hepatitis/hepatitis-b-guidelines/en/).

The outcomes of HBV infection depend on both viral and host factors, including genetic factors that determine a host’s immune mechanisms [3]. It is generally accepted that HBV is not directly cytopathic; thus, HBV-related hepatocellular injuries are the results of the complex interplay among HBVs, hepatocytes, and host immune cells [3, 4]. It has been widely recognized that host immunity contributes to the pathogenesis of liver injuries and even liver failure. The immune clearance of HBV can trigger extensive liver damage that results in fibrosis and cirrhosis. Cytotoxic T-lymphocytes (CTLs), the core of cellular immunity, play a key role in the clearance of intracellular viruses, which are the major cause of cell apoptosis or necrosis [5]. Several studies have characterized intrahepatic CD4^+^ and CD8^+^ T lymphocytes in chronic hepatitis B (CHB) (as reviewed by Shimizu) [6]. It is generally accepted that HBV-specific CD8^+^ T cells have important functions in controlling HBV replication in the liver without causing hepatic necroinflammation, whereas non-specific CTLs may contribute to HBV-related intrahepatic inflammation [7].

Macrophages Cells play a key role in the homeostasis of the liver, which undergo polarized activation to M1 or M2 or M2-like activation states in response to environmental signals. The M1 phenotype is characterized by the upregulation of proinflammatory cytokines and promotion of Th1 response, and strong microbicidal and tumoricidal activity. In contrast, M2 macrophages promote tissue remodeling and tumor progression, and have immunoregulatory functions (as reviewed by Antonio Sica, et al.) [8]. It is general accepted that HBV promotes intrahepatic resident and recruited macrophages M2 polarization, leading to impairment of host immunity and progression of tissue fibrosis/remodeling [9].

Th cells that produce IL-17 (Th17 cells) have recently been identified as the third subset of effector T cells, which produce IL-17A, IL-17 F, IL-22 and IL-21 [10]. High numbers of IL-17-producing CD4^+^ T cells have been observed in both the liver and the blood of CHB patients; and the elevation in this cell population has been correlated with a high serum level of IL-27 [11]. An increase in circulating and intrahepatic IL-17-producing CD4^+^ T cells is well correlated with ALT level and liver injury. CD4^+^ T lymphocytes that produce IL-17 infiltrate into the livers of patients with CHB and increase the severity of liver damage [12].

Prior studies on HBV-related hepatitis flares have demonstrated that, high ALT levels usually accompanied by increased serum levels of IL-12 and Th1 phenotypic cytokines (IL-2 and IFN-γ) [13, 14], and the natural killer (NK)-cell mediated liver damage were found to be attributed to the increased serum IFN-a and IL-8 [15]. Therefore, hepatitis B flares are results of complex interplay of the virus and the innate and adaptive immune responses, and the more vigorous immune response against HBV, the higher the serum ALT (as reviewed by Chang and Liaw) [4].

Hepatic microcirculation disorders occur in all chronic liver diseases, which result in insufficient blood supply to hepatocytes. In addition, collateral circulation also depletes blood flow from the liver. As a result, a) nutrients absorbed from the intestines cannot nourish the liver; b) the therapeutic effects of certain drugs are decreased; and c) metabolic wastes accumulate. These events speed the progression of liver diseases.

Resistin is a 12.5-kd adipokine that belongs to a new family of small, cysteine-rich secretory proteins known as FIZZ (found in inflammatory zone) or resistin-like molecules [16]. In rodents, resistin is highly expressed in adipose tissue, and circulating levels of resistin are increased during diet-induced or genetic obesity [17]. It has been verified that resistin is expressed and upregulated under conditions of chronic injury in human liver tissue, and resistin can stimulate human hepatic stellate cells (HSCs) to secrete proinflammatory cytokines through activating the nuclear factor (NF)-κB signaling pathway [18]. In patients with chronic hepatitis C virus infection, low serum levels of resistin are associated with the presence of fibrosis and may therefore be a biochemical marker of fibrosis [19]. Moreover, Tsochatzis et al. found that in CHB and chronic hepatitis C (CHC) patients, resistin levels are independently associated with fibrosis severity [20]. Another study found that elevated levels of resistin were prominent in patients with hepatobiliary inflammation and were associated with breach of self-tolerance; thus, resistin may be an important marker of disease severity in autoantibody-mediated gastrointestinal inflammatory diseases [21]. Furthermore, increased serum resistin is known to be positively correlated with histological inflammatory score in nonalcoholic fatty liver disease (NAFLD), suggesting that increased resistin in NAFLD patients is related to the histological severity of this disease [22].

In the present study, patients with chronic HBV infection were retrospectively selected. The serum resistin levels and serum levels of the cytokines IL-1, IL-6, IL-17, IL-23, TNF-α, and TGF-β1 were assayed. The association between serum resistin levels and serum cytokine levels, liver biochemical indices, and fibrosis severity were analyzed. The possible role of resistin as a marker of the inflammatory process in patients with CHB was investigated in detail.

## Methods

### Patients

Patients with CHB were retrospectively selected from Aug. 2013 to Sept. 2014 at the Inpatient Department of Taihe Hospital, Hubei University of Medicine. CHB, HBV-related liver cirrhosis (LC-B), and HBV-related liver failure (LF-B) were diagnosed in accordance with published guidelines [23, 24]. Patients’ serum samples were routinely stored and used for this retrospective study. The inclusion criteria were chronic infection with HBV, defined as detectable HBsAg and HBV-DNA for at least 6 months, and age ≥ 18 years. Patients were excluded if they were co-infected with the hepatitis A virus (HAV), hepatitis C virus (HCV), hepatitis D virus (HDV), hepatitis E virus (HEV), or human immunodeficiency virus (HIV); if they reported consuming significant quantities of alcohol (more than 30 g per week for men and 20 g per week for women); if they had received a liver allograft; or if a malignant disease, including HCC, had been diagnosed. Clinical databases were consulted to obtain patients’ demographic and clinical characteristics, including age; sex; serum levels of alanine aminotransferase (ALT), aspartate aminotransferase (AST), and total bilirubin (TBil); and liver stiffness (LS). The study protocol was approved by the Ethics Committee of Taihe Hospital,Hubei university of Medicine. Written informed consent was given by all the participants prior to their inclusion in this study. All data and samples used were collected during standard clinical care. Twenty serum samples from blood donors were used as healthy controls (HCs).

### Quantification of serum levels of resistin

Serum levels of resistin were measured using ELISA kits (eBioscience, USA) according to the manufacturer’s instructions (with resistin sensitivity < 3.1 pg/mL).

### Quantification of serum levels of IL-1, IL-6, IL-17, IL-23, TNF-α and TGF-β1

Serum levels of IL-1, IL-6, IL-17, IL-23, TNF-α, and TGF-β1 were measured using ELISA kits (R&D Systems, USA) in accordance with the manufacturer’s instructions. Standard curves were constructed using standard samples (IL-1β sensitivity < 1 pg/mL; IL-6 sensitivity < 0.7 pg/mL; IL-17 sensitivity < 15 pg/mL; IL-23 sensitivity < 16.3 pg/mL; TNF-α sensitivity < 5.5 pg/mL; and TGF-β1 sensitivity < 15.4 pg/mL).

### Statistical analyses

Study data are presented as means ± SD. Variables were compared using a general linear model, Student’s *t*-test, or the Mann–Whitney *U* test as needed. Statistical analysis was performed using SPSS for Windows. Simple linear correlation analyses were conducted using Pearson’s method to assess the correlations between resistin and cytokines, AST, and TBil. The threshold used for statistical significance was *p* < 0.05. The statistical methods of this study were reviewed by Dr. Jing Wang from the Department of Epidemiology and Hygenic statistics, Hubei Univeristy of Medicine.

## Results

### Patient characteristics

After applying the criteria described above, 234 patients were selected for the present study: 85 patients with CHB, 70 patients with LC-B, and 79 patients with LF-B. Baseline characteristics of these patients are summarized in Table 1. The male/female ratios for CHB patients, LC-B patients, and LF-B patients were 64/21, 42/28, and 65/14, respectively. Most patients received NA-based antiviral treatment, in which entecavir is most used, except that some CHB patients received IFN-α treatment. Serum levels of ALT, AST, and TBil were 178.894 ± 205.229 IU/L, 116.865 ± 146.940 IU/L, and 41.843 ± 72.044 mmol/L, respectively, for CHB patients; 61.043 ± 117.280 IU/L, 70.171 ± 114.080 IU/L, and 38.336 ± 43.166 mmol/L, respectively, for LC-B patients; and 86.861 ± 270.105 IU/L, 172.730 ± 219.91 IU/L, and 238.420 ± 139.550 mmol/L, respectively, for LF-B patients (Table 2). The primary analyses of this study focused on serum levels of resistin, IL-1, IL-6, IL-17, IL-23, TNF-α and TGF-β1, which were generally determined using serum samples obtained upon patients’ initial presentation in the Department of Infectious Diseases, Taihe Hospital, Hubei University of Medicine.

**Table 1:** Clinical characteristics of the enrolled patients

Demographics	CHB (*n* = 85)	LC-B (*n* = 70)	LF-B (*n* = 79)
Male/female	64/21	42/28	65/14
Age (years), mean ± SD	39.800 ± 14.900	48.330 ± 11.050	46.400 ± 10.000
HBeAg positive/negative	47/38	26/44	30/49
HBV DNA (log10) IU/ml	6.70 ± 1.45	6.06 ± 1.17	6.45 ± 1.35
Antivirus treatment (Y/N)	62/23	56/14	68/11
ALT (IU/L)	178.894 ± 205.229	61.043 ± 117.280	186.861 ± 270.105
AST (IU/L)	116.865 ± 146.940	70.171 ± 114.080	172.730 ± 219.91
TBil (mmol/L)	41.843 ± 72.044	38.336 ± 43.166	238.420 ± 139.550

**Table 2:** Serum levels of resistin and cytokines

Groups	HC	CHB	LC-B	LF-B
Resistin (ng/mL)	0.078 ± 0.270	4.119 ± 5.848	6.370 ± 6.834	6.512 ± 6.076
IL-1 (pg/mL)	0.077 ± 0.186	0.549 ± 1.341	0.932 ± 1.754	0.446 ± 1.104
IL-6 (pg/mL)	0.077 ± 0.186	8.830 ± 19.426	21.822 ± 50.372	29.792 ± 51.394
IL-17 (pg/mL)	2.923 ± 2.310	5.410 ± 5.634	5.164 ± 3.522	5.288 ± 5.860
IL-23 (pg/mL)	4.589 ± 3.823	8.149 ± 17.379	6.103 ± 12.005	5.874 ± 10.981
TNF-α (pg/mL)	2.489 ± 2.083	9.038 ± 8.108	27.961 ± 120.362	43.472 ± 145.516
TGF-β1 (pg/mL)	29.380 ± 3.339	46.205 ± 7.818	48.636 ± 11.555	55.537 ± 6.971

### Patients with chronic HBV infection had significantly elevated serum resistin levels

**Fig. 1:**
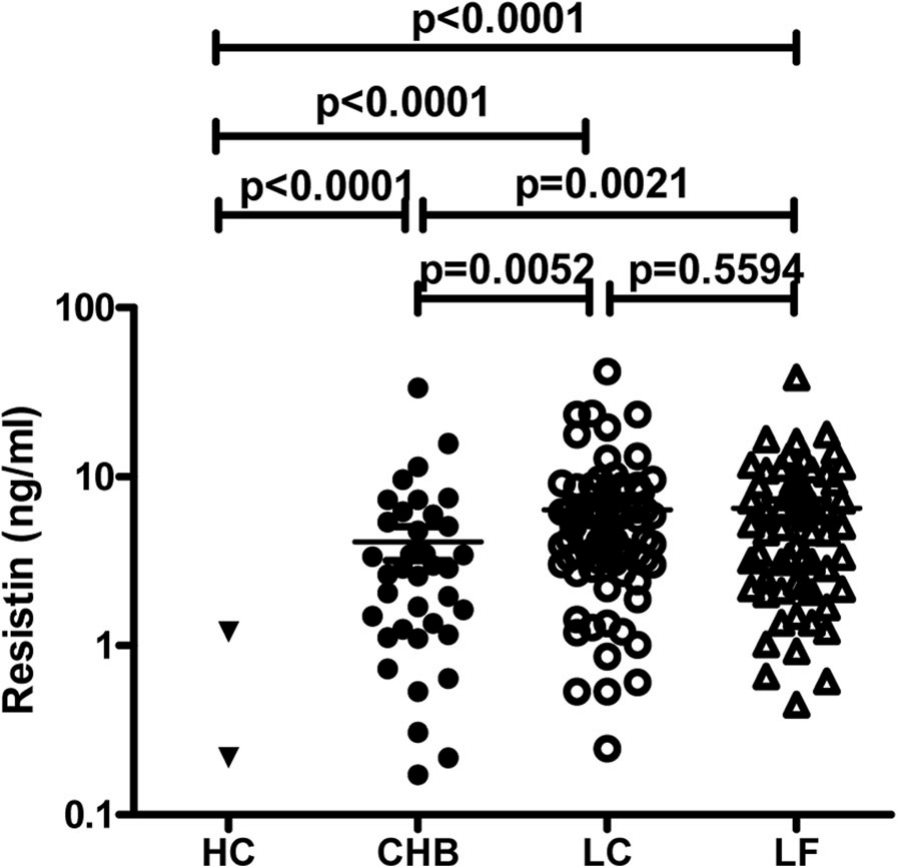
Serum levels of resistin in patients with HBV infection. Serum resistin levels were assayed by ELISA and analyzed using GraphPad software. Differences between different groups and HCs were assessed using the Mann–Whitney test. HC, healthy control; CHB, chronic hepatitis B; LC, liver cirrhosis; LF, liver failure

Serum resistin was rarely detectable in the HC group (0.078 ± 0.270). In contrast, high serum resistin levels were detected in patients with CHB (4.119 ± 5.848), LC-B (6.370 ± 6.834), and LF-B (6.512 ± 6.076) (Table 1, Fig. 1). Compared with CHB patients, LC-B patients and LF-B patients had significantly higher serum levels of resistin (*p* < 0.01), whereas LC-B patients and LF-B patients did not significantly differ with respect to serum resistin levels (*p* > 0.05) (Fig. 1).

### Patients with chronic HBV infection had significantly increased serum levels of IL-1, IL-6, IL-17, TNF-α, and TGF-β1, but not IL-23

**Fig. 2:**
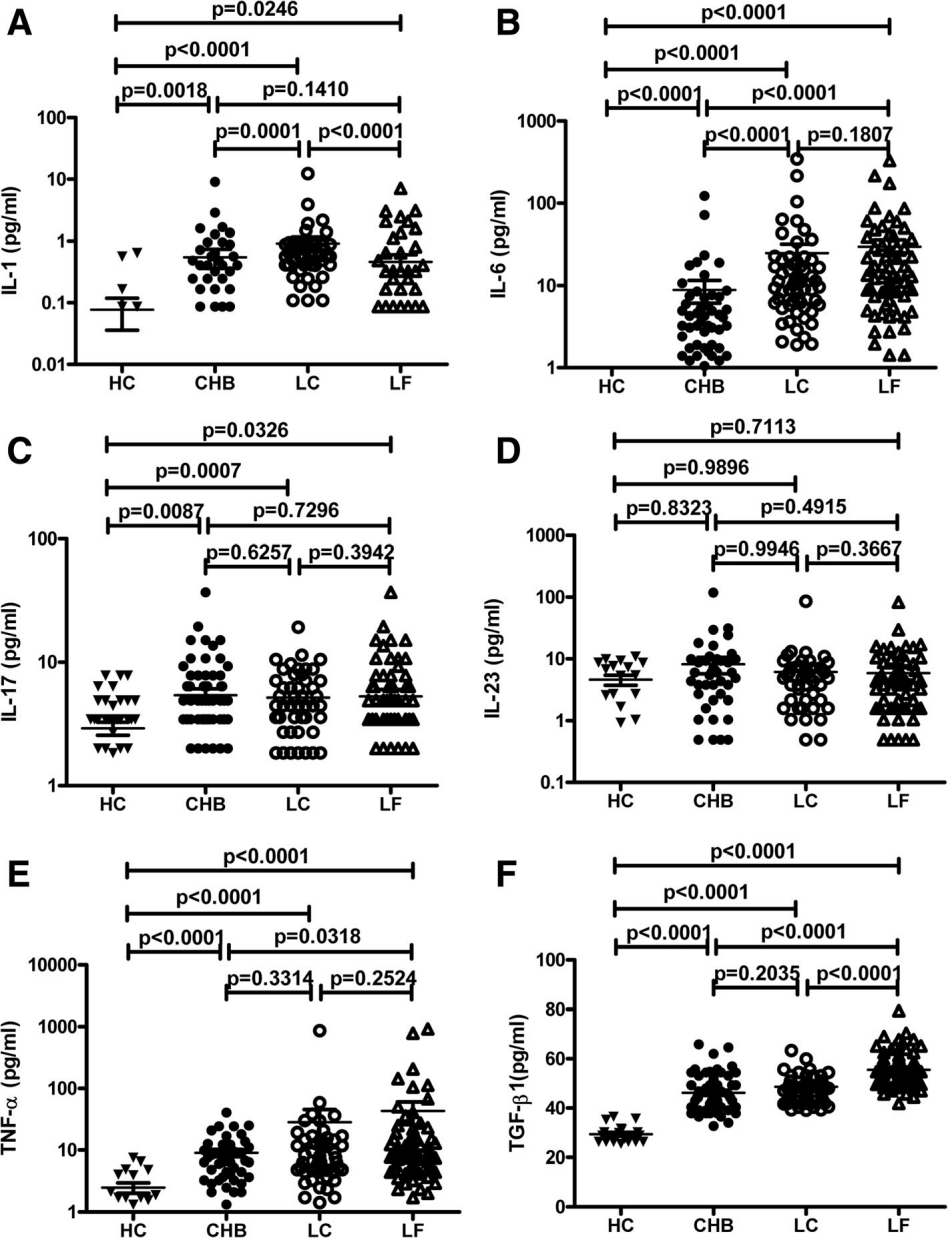
Serum levels of IL-1, IL-6, IL-17, IL-23, TNF-α, and TGF-β1 in patients with HBV infection. Serum levels of IL-1 (**a**), IL-6 (**b**), IL-17 (**c**), IL-23 (**d**), TNF-α (**e**), and TGF-β1 (**f**) were assayed by ELISA and analyzed using GraphPad software. Differences between different groups and HCs were assessed using the Mann–Whitney test. HC, healthy control; CHB, chronic hepatitis B; LC, liver cirrhosis; LF, liver failure

With respect to cytokine detection, serum IL-1 and IL-6 were below the detection limit in HCs, whereas IL-17 (2.923 ± 2.310 pg/mL), IL-23 (4.589 ± 3.823 pg/mL), TNF-α (2.489 ± 2.083 pg/mL), and TGF-β1 (29.380 ± 3.339 pg/mL) were detected in these subjects. Compared with HCs, patients with CHB, LC-B, or LF-B had elevated levels of IL-1, IL-6, IL-17, TNF-α, and TGF-β1 (Table 1, Fig. 2). Serum IL-1 levels were higher in LC-B patients (0.932 ± 1.754 pg/mL) than in CHB patients (0.549 ± 1.341 pg/mL) and LF-B patients (*p* < 0.001) (Fig. 2a). LC-B patients (21.822 ± 50.372 pg/mL) and LF-B patients (29.792 ± 51.394 pg/mL) had markedly higher serum IL-6 levels than CHB patients (*p* < 0.001), whereas the serum IL-6 levels of LC-B patients and LF-B patients did not significantly differ (*p* > 0.05) (Fig. 2b). All patients had high serum IL-17 and TNF-α levels, with no significant differences in these cytokines among CHB patients, LC-B patients, and LF-B patients (*p* > 0.05) (Fig. 2c and e). Serum TGF-β1 levels were higher in LF-B patients (55.537 ± 6.971 pg/mL) than in CHB patients (46.205 ± 7.818 pg/mL) and LC-B patients (48.636 ± 11.555 pg/mL) (*p* < 0.001) (Fig. 2f). Average serum IL-23 levels were higher for patients than for HCs but did not significantly differ among CHB patients, LC-B patients, and LF-B patients (*p* > 0.05) (Fig. 2d).

### Serum resistin was positively correlated with serum IL-17 among patients with LC-B or LF-B

**Fig. 3:**
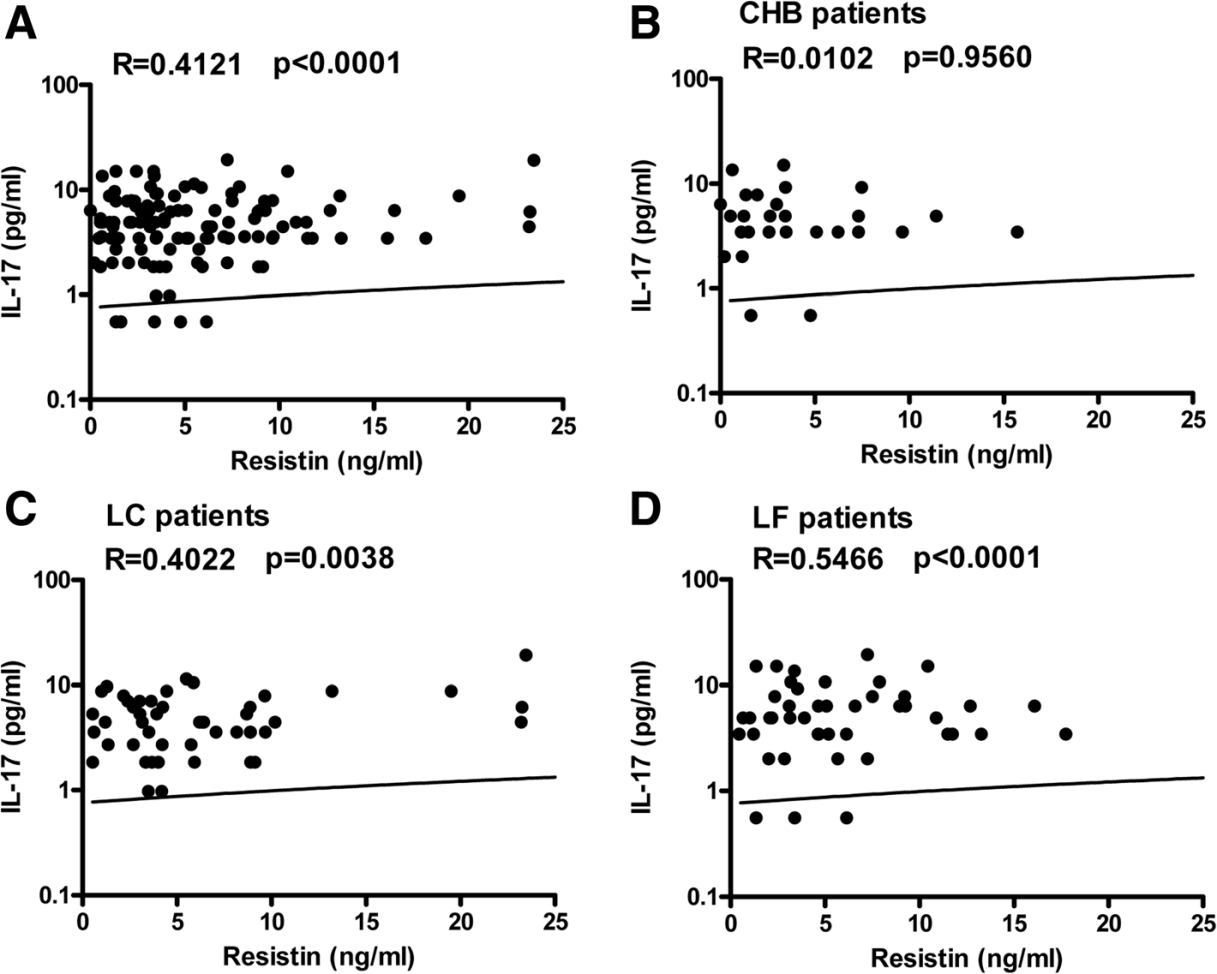
The relationship between serum resistin and serum IL-17. Serum resistin levels and serum IL-17 levels were analyzed by correlation analysis using GraphPad software. The correlations between resistin levels and serum IL-17 levels among all hepatitis B patients (**a**), CHB patients (**b**), LC-B patients (**c**), and LF-B patients (**d**) are presented. CHB, chronic hepatitis B; LC, liver cirrhosis; LF, liver failure

Among all patients with chronic HBV infection, serum resistin was positively correlated with serum IL-17 (*R* = 0.4121, *p* < 0.0001) (Fig. 3a). Further analysis demonstrated that serum resistin was strongly positively correlated with serum IL-17 among LF-B patients (*R* = 0.5466, *p* < 0.0001) (Fig. 3d), more weakly positively correlated with serum IL-17 among LC-B patients (*R* = 0.4022, *p* = 0.0038) (Fig. 3c), and not correlated with serum IL-17 among CHB patients (*R* = 0.0102, *p* = 0.9560) (Fig. 3b).

### Serum resistin was positively correlated with serum TGF-β1 in patients with LC-B

**Fig. 4:**
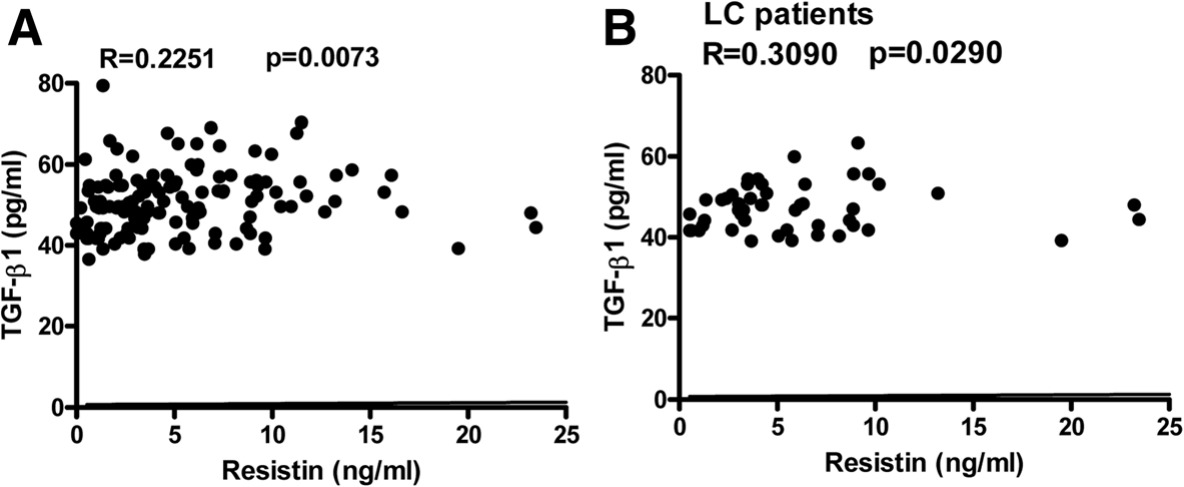
The relationship between serum resistin and serum TGF-β1. Serum resistin levels and serum TGF-β1 levels were analyzed by correlation analysis using GraphPad software. The correlations between resistin levels and serum TGF-β1 levels among all hepatitis B patients (**a**) and LC-B patients (**b**) are presented. LC, liver cirrhosis

Subsequently, the relationship between serum resistin and serum TGF-β1 was analyzed. Positive correlations between serum resistin and serum TGF-β1 were observed for all patients with chronic HBV infection (*R* = 0.2251, *p* = 0.0073) (Fig. 4a). Subgroup analysis indicated that serum resistin was weakly positively correlated with serum TGF-β1 among LC-B patients (*R* = 0.3090, *p* = 0.0290) (Fig. 4b). Serum resistin was not correlated with serum TGF-β1 among CHB patients and LF-B patients (data not shown).

### Serum resistin levels were positively correlated with LS in patients with CHB

**Fig. 5:**
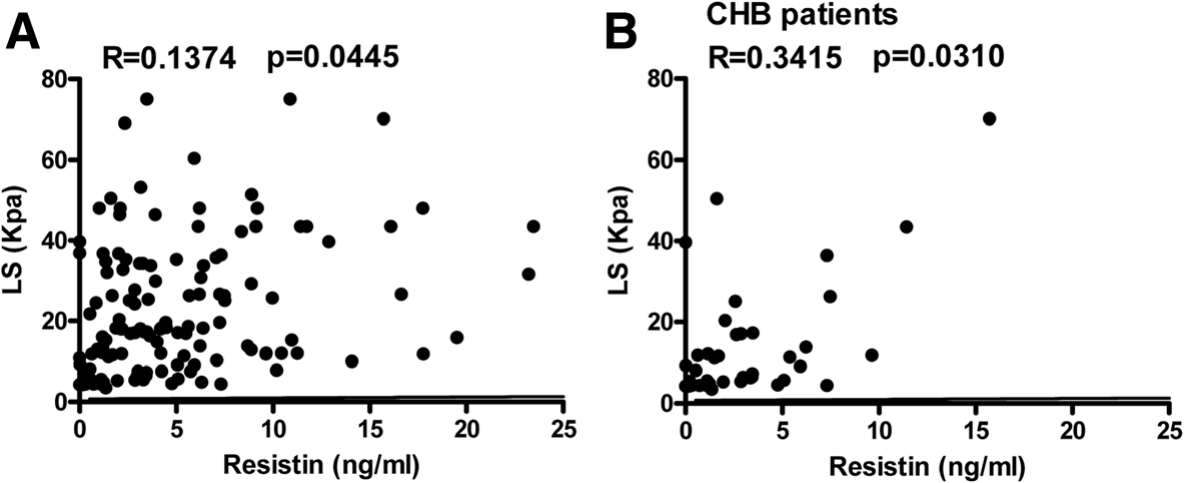
The relationship between serum resistin and LS. Serum resistin levels and LS values were analyzed by correlation analysis using GraphPad software. The correlation between resistin levels and LS among all hepatitis B patients (**a**) and CHB patients (**b**) are presented. CHB, chronic hepatitis B

The relationship between serum resistin levels and LS was also analyzed. A weak positive correlation with LS was found among all patients with HBV infection (*R* = 0.1374, *p* = 0.0445) (Fig. 5a). In subgroup analysis, this correlation was only found among CHB patients (*R* = 0.3415, *p* = 0.0310) (Fig. 5b). Serum resistin levels were not correlated with LS among LC-B patients or LF-B patients (data not shown).

### Serum resistin was positively correlated with serum AST in patients with CHB

**Fig. 6:**
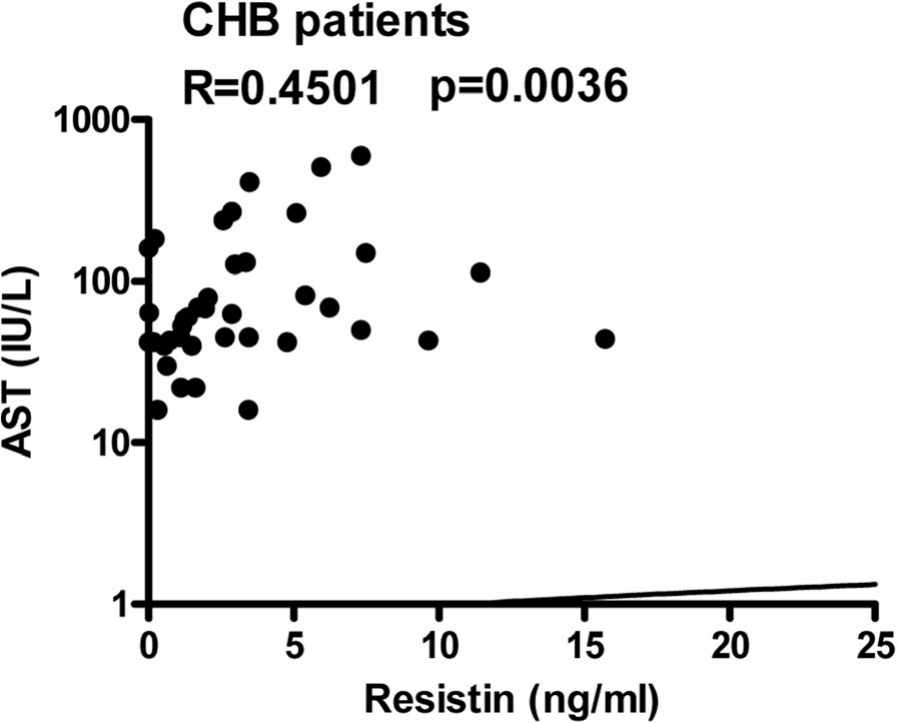
The relationship between serum resistin and serum AST. Serum resistin levels and serum AST levels were analyzed by correlation analysis using GraphPad software. The correlation between resistin levels and serum AST levels among CHB patients is presented. CHB, chronic hepatitis B

The relationships between serum resistin and serum ALT, AST, and TBil were also analyzed. A positive correlation between serum resistin and serum AST was observed among CHB patients (*R* = 0.4501, *p* = 0.0036) (Fig. 6). Serum resistin was not correlated with serum AST among LC-B patients or LF-B patients; in addition, no correlations between serum resistin and serum ALT or TBil were detected either among all enrolled patients or in any examined subgroup (data not shown).

## Discussion

This study revealed that patients with chronic HBV infection had significantly elevated serum resistin levels; this finding is consistent with previously reported data [20]. Patients with LC-B or LF-B had significantly higher serum resistin levels than CHB patients (*p* < 0.01), whereas LC-B patients and LF-B patients did not significantly differ with respect to serum resistin levels (*p* > 0.05). These results suggest that serum resistin could play a role as an indicator of disease severity and/or degeneration in patients with hepatitis B. Moreover, high serum levels of IL-1, IL-6, IL-17, TNF-α, and TGF-β1 but not IL-23 were detected in CHB, LC-B, and LF-B patients. Serum IL-1 levels were higher in LC-B patients than in CHB and LF-B patients, whereas serum IL-6 and TNF-α levels were much higher for LC-B patients and LF-B patients than for CHB patients; these findings are consistent with the inflammatory roles of the proinflammatory cytokines IL-1, IL-6, and TNF-α [25].

In this study, serum resistin levels were weakly correlated with LS values determined by FibroScan among CHB patients but not among LC-B patients or LF-B patients. This result can be explained by the fact that LS depends on the extent of fibrosis due to prior intrahepatic deposits [26]. Serum markers such as procollagen peptide, matrix metalloproteinases (MMPs), tissue inhibitors of matrix metalloproteinases (TIMPs), laminins, and TGF-β1, are correlated with molecules derived directly from the ECM or produced by activated HSCs. Thus, the elevation of these serum markers suggests the activation of fibrogenesis [27, 28]. Interestingly, serum resistin levels were positively correlated with serum TGF-β1 levels, particularly among LC-B patients (Fig. 4). Furthermore, among CHB patients, serum resistin levels were positively correlated with serum AST levels (Fig. 5). These results are consistent with the general notion that resistin can act as an intrahepatic cytokine with proinflammatory activity in HSCs via a Ca2^+^/NF-κB-dependent pathway and involvement in the pathophysiology of liver fibrosis [18].

IL-17 is a major effector cytokine secreted by Th17 cells, which play a proinflammatory role in the pathogenesis of hepatitis B and promote HBV infection-related injury [29]. In this study, all patients with CHB, LC-B, or LF-B exhibited similarly elevated serum IL-17 levels (Fig. 2c). There were no significant correlations between serum IL-17 and serum ALT, AST, or TBil (data not shown). Serum IL-17 was positively correlated with serum resistin among LC-B patients and LF-B patients but not among CHB patients (Fig. 3); these findings provide additional evidence supporting the proinflammatory role of IL-17, especially in the context of advanced liver injury.

Recently, Ming et al. found that the upregulation of the TGF-β1/IL-31 pathway is associated with disease severity in LC-B, since elevated serum TGF-β1 and IL-31 levels were positively associated with albumin, alpha-fetoprotein, creatinine, white blood cell, and platelet levels among LC-B patients [30]. Furthermore, TGF-β1/IL-31 pathway may also play an important role in the pathogenesis of liver injury during chronic HBV infection, since increased activity of the TGF-β1/IL-31 pathway has been found well correlated with the extent of liver injury, disease severity, and nonsurvival among ACLF patients, whereas reduced activity of this pathway has been detected during the recovery from liver injury in CHB cases [31]. In the current study, patients with HBV chronic infection exhibited elevated serum TGF-β1, and serum TGF-β1 levels were significantly higher in LF-B patients than in CHB patients or LC-B patients. CHB and LC-B patients did not significantly differ with respect to serum TGF-β1, although average serum TGF-β1 levels were slightly higher in LC-B patients than in CHB patients. These results provided additional data to support the potential role of TGF-β1 in the pathogenesis of liver injury in patients with chronic HBV infection, particularly patients with LF-B.

IL-23 has recently been identified as playing a critical role in a number of chronic inflammatory diseases. Xia et al. reported that both serum IL-23 level and hepatic IL-23 were positively correlated with liver injury in CHB patients [32]. In this study, average serum IL-23 levels were higher in patients than in HCs and were markedly higher in CHB patients than in HCs (8.149 vs. 4.589), with no significant differences in serum IL-23 levels among CHB patients, LC-B patients, and LF-B patients. This lack of significance may be attributable to the large deviations detected in serum IL-23 levels (which ranged from 0.49 to 118.92).

Taken together, the findings of this study demonstrated that high serum resistin was positively correlated with serum IL-17 and TGF-β1. Elevated resistin is associated with the inflammation and necrosis of liver cells and could therefore potentially be used as an index of disease severity and degeneration in patients with chronic HBV infection. However, the mechanism of resistin in the inflammatory process of chronic hepatitis B is unclear, further studies are needed to elucidate how resistin works in the progress of liver injury, and the cross-talk between resistin and IL-17 or TGF-β signaling pathways.

## Conclusions

High serum resistin associates with intrahepatic inflammation and necrosis and may be used as an index of disease severity for patients with chronic HBV infection.

## Additional file

Additional file 1: The raw data of laboratory tests for serum levels of resistin, IL-1, IL-6, IL-17, IL-23, TNF-α, TGF-β1, ALT, AST and TBil. (XLS 157 kb)

## Abbreviations

ALT: Alanine aminotransferase
AST: Aspartate aminotransferase
CHB: Chronic hepatitis B
CTL: Cytotoxic T-lymphocyte
ELISA: Enzyme-linked immunobsorbent assay
HBV: Hepatitis B virus
HSC: Hepatic stellate cells
LC: Liver cirrhosis
LF: Liver failure
LS: Liver stiffness
NAFLD: Nonalcoholic fatty liver disease
TGF-β: Transforming growth factors beta
TNF-α: Tumor necrosis factor alpha
